# Impact of maternal nutritional supplementation in conjunction with a breastfeeding support program during the last trimester to 12 weeks postpartum on breastfeeding practices and child development at 30 months old

**DOI:** 10.1371/journal.pone.0200519

**Published:** 2018-07-16

**Authors:** Zhiying Zhang, Nga T. Tran, Tu S. Nguyen, Lam T. Nguyen, Yatin Berde, Siew Ling Tey, Yen Ling Low, Dieu T. T. Huynh

**Affiliations:** 1 Abbott Nutrition Research and Development Asia-Pacific Center, Singapore; 2 National Institute of Nutrition, Ha Noi, Vietnam; 3 Statistical Services, Cognizant Technologies Solution Pvt. Ltd., Airoli, Navi Mumbai, India; TNO, NETHERLANDS

## Abstract

**Background:**

Maternal nutrition during pregnancy and breastfeeding is important for the healthy growth and development of the fetus and infant.

**Purpose:**

This study aimed to evaluate the long-term effects of a maternal milk supplementation (MMS) in conjunction with a breastfeeding support program on breastfeeding practices including duration of any breastfeeding and exclusive breastfeeding and child neurodevelopment outcomes at 30 months old.

**Methods:**

We followed up the offspring of 204 Vietnamese women who completed a randomized controlled trial where the intervention group received MMS with a breastfeeding support program from the last trimester to 12 weeks postpartum while the control group received standard care. At 30 months postpartum, information on child feeding practices was collected and child neurodevelopment was assessed by the Bayley Scales of Infant and Toddler Development (Bayley-III).

**Results:**

There was no significant difference in the duration of any breastfeeding (ABF) from birth between the groups. However, the intervention group had longer exclusive breastfeeding (EBF) duration (p = 0.0172), higher EBF rate at 6 months (p = 0.0093) and lower risk of discontinuing EBF (p = 0.0071) than the control. Children in the intervention group had significantly higher Bayley-III composite scores in the domains of cognitive (p = 0.0498) and motor (p = 0.0422) functions, as well as a tendency toward better social-emotional behavior (p = 0.0513) than children in the control group. The association between maternal intervention and child development was attenuated after further adjustment for birth weight but not EBF duration, suggesting that improvements in child development may be partially attributed to the benefits of prenatal nutrition supplementation on birth outcomes.

**Conclusions:**

MMS with breastfeeding support during late pregnancy and early postpartum significantly improved EBF practices. The intervention was also associated with improvements in neurodevelopment in children at 30 months old.

## Introduction

Optimal maternal nutrition during pregnancy and breastfeeding is critical for the healthy growth and development of the fetus and infant, which lays the foundation for their long-term health in later life. However, poor nutritional status in both mother and her child remains prevalent in low- and middle-income countries [1,2]. Since pregnancy and lactation represent about half of the “first 1000 days” window, improving maternal nutrition to support healthy birth outcomes and successful breastfeeding in these settings has the potential to improve the life-long health of the offspring.

Numerous studies have shown positive effects of maternal supplementation during pregnancy and breastfeeding on maternal, fetal, infant and child health outcomes [3,4,5,6,7]. These include reducing the risk of birth defects [8,9], increasing birth weight, length and head circumference [5,10,11,12], raising exclusive breastfeeding (EBF) rates [13] and promoting neurodevelopment [6]. Yang and Huffman reviewed nutrition intervention trials for pregnant and lactating women and categorized the studies according to different types of fortified foods and products, including protein/fat free fortified beverages, products containing cow’s milk, fortified high-fat products, and formulated non-milk drinks [3]. When both maternal nutritional status improvements and birth outcome improvements are the focus of interest, fortified food supplements such as those containing milk and/or essential fatty acids, are preferred over fortified beverages containing only micronutrient[3]. Given that requirements of many nutrients, both macro- and micronutrients, are increased during pregnancy and lactation [14,15,16], providing a supplement with a full suite of macro- and micronutrients specifically designed for pregnant and lactating women may provide greater benefits. Although a few recent trials have shown positive effects of lipid-based nutrient supplements (LNS) in pregnant and lactating women and their infants [17], the present study focuses on research on the type of products containing cow’s milk [3]. Given that requirements of many nutrients, both macro- and micronutrients, are increased during pregnancy and lactation [14,15,16], providing a supplement with a full suite of macro- and micronutrients specifically designed for pregnant and lactating women may provide greater benefits. Although a few recent trials have shown positive effects of lipid-based nutrient supplements (LNS) in pregnant and lactating women and their infants [17], the present study focuses on research on the type of products containing cow’s milk. [17]

While relatively more studies have evaluated the short-term outcomes of maternal supplementation, research is limited on the long-term effects including postnatal growth and development in the offspring [18,19]. Few intervention trials involving maternal protein-energy supplementation were conducted in different regions of the world [20], including Guatemala [21], Mexico [22], Bogota, Colombia [23], Taiwan [24], New York City [25] and more recently Hyderabad, India [26] and the West Kiang region of the Gambia [27]. Among these three trials have investigated the long-term effects of maternal supplementation without supplementing the child, all of which were conducted between the 1960s and 1980s [24,25,28,29]. Although previous studies had made efforts to account for a few confounding factors such as the child’s age and gender, several important factors influencing child development, for example the child’s nutritional status, sociodemographics, and home environment were not considered in the multivariate analyses evaluating the effects of maternal supplementation on child growth and development. Given that these studies were conducted a few decades ago, an updated study which takes into account the effects of potential confounders would provide a more comprehensive view on the role of maternal supplementation during the “first 1000 days” window in long-term health benefits of the offspring.

We recently conducted a randomized controlled trial to evaluate the effects of maternal milk supplementation(MMS) in conjunction with a breastfeeding support program in the last trimester until 3 months postpartum on birth outcomes, short-term breastfeeding practices and child growth in comparison to the standard care in Vietnamese mothers [30]. The findings showed that the intervention during the last trimester significantly improved birth weight and birth head circumference compared with the control. Infants in the intervention group also had significantly higher EBF rate and growth of head circumference during the first 3 postnatal months than those in the control group. To our knowledge, there is limited research on the sustained effects of MMS in combination with breastfeeding education and support on post-intervention breastfeeding practice and the child’s growth and development. Thus, we conducted this observational follow-up study to evaluate longer-term breastfeeding practices, growth and neurodevelopmental functions in children at 2.5 years in consideration of potential confounding and mediating factors of the child development. We hypothesized that supplementing mothers with a nutrient-dense MMS during the last trimester of pregnancy and early lactation period as part of a breastfeeding program may have sustained positive effects on breastfeeding and child’s growth and neurodevelopment.

## Methods

### Study design and participants

This was an observational study involving mothers and their children at 30 months old as a follow-up to a prospective, randomized, open-label, parallel-group, multicenter trial that they participated in. The study design and participants’ characteristics of the randomized controlled trial (RCT) have been described in detail previously [30].

**Fig 1:**
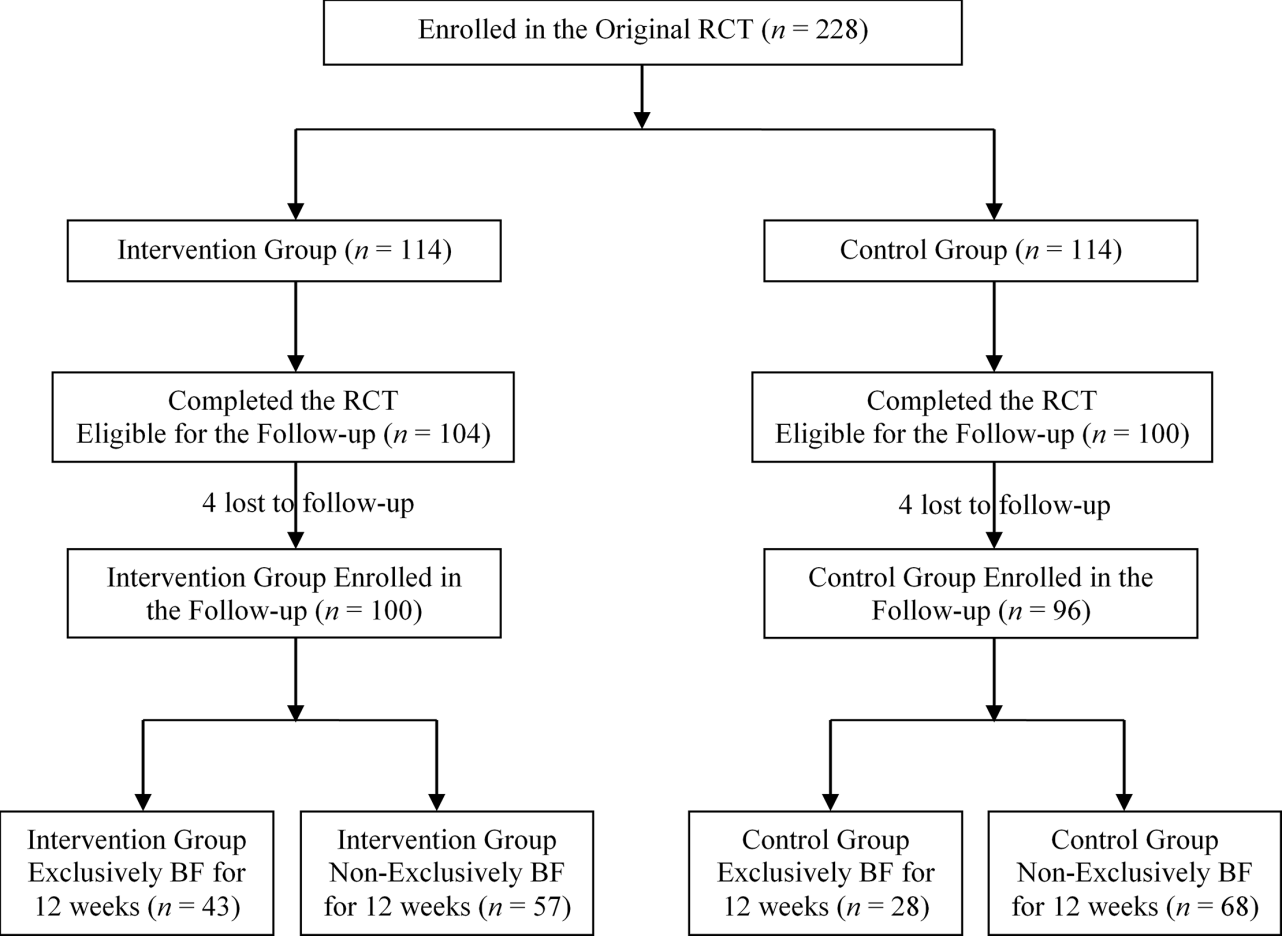
Study flow chart. RCT, randomized controlled trial; BF, breastfed.

In brief, a total of 228 singleton Vietnamese mothers aged 21 to 35 years at 26 to 29 weeks of gestation with a pre-pregnancy body mass index (BMI) <25 kg/m^2^ took part in the RCT across four northern provinces in Vietnam including Ninh Binh, Ha Nam, Hai Phong, and Thai Nguyen. Mothers who were randomized to the intervention (n = 114) received two daily servings of MMS (which provided 252 kcal, 16.8 g protein, 1.4 g fat, 39.2 g carbohydrate, and a variety of micronutrients) up to 12 weeks postpartum and 4 breastfeeding education and support sessions [30]. The control group (n = 114) received standard antenatal care per local health policy, including continued supplementation from enrollment to delivery with folic acid (400 mcg) and iron (60 mg) and breastfeeding advice during prenatal visits only if this was part of the standard of care at the sites. At the end of the intervention (12 weeks postpartum), 104 and 100 mother-infant pairs in the intervention and control group respectively completed the RCT (Fig 1).

When the children were about 30 months old, all mother and child pairs who completed the RCT were invited to participate in this observational study. Children with a diagnosis of severe developmental disorders e.g. cerebral palsy which required medical intervention were excluded from this study. Written informed consent was obtained from each mother and the study was approved by the Independent Ethics Committees of the National Institute of Nutrition (NIN) and the Ministry of Health in Vietnam. The original trial and the present study were registered at clinicaltrials.gov with registration codes NCT02016586 and NCT02913638, respectively [31].

### Maternal breastfeeding and child feeding practices

The primary outcome was duration of any breastfeeding (ABF) from birth. At 30 months postpartum, the mothers were asked to fill out a questionnaire, which was used to capture information on the age of the child (in months/weeks) in which the mother stopped breastfeeding. In addition, the questionnaire also captured information on the age of the child (in months/weeks) in which the mother introduced non-breast milk substitutes including infant formula and liquids such as water or intake of solids in mothers who had exclusively breastfed from birth to 12 weeks postpartum. For mothers who had stopped EBF by 12 weeks postpartum, dietary record information of the infant’s intake of breast milk and non-breast milk including formula, juice, water and complementary foods collected during the intervention study was used to assess EBF from birth. The World Health Organization (WHO) definition of exclusive breastfeeding “the infant receives breast milk as the only source of nutrition (including milk expressed or from a wet nurse), with no other liquids or solids except for oral rehydration solution, vitamin drops, minerals and medicines” was used in this study [32]. Any breastfeeding was defined as the child being exclusively/predominantly breastfed or receiving both breast milk and formula(s), regardless of whether other breast-milk substitutes or solids had been given. Breastfeeding duration was defined as the age of the child at which breastfeeding ceased.

Child feeding practices after completion of the RCT through the first year included timing of introduction of semi-solids or solid foods, and practice of fortifying child’s food using vegetable oil during food preparation.

### Child neurodevelopment assessment

Neurodevelopment of the children was measured using two validated tools, namely the Ages and Stages Questionnaire, Third Edition (ASQ-3) and the Bayley Scales of Infant and Toddler Development, Third Edition (Bayley-III). ASQ-3 is a parent-completed, child-development screening test that assesses the child’s communication, gross and fine motor, problem solving and personal social developments. Bayley-III is a widely recognized comprehensive tool to assess the development of children aged 1 to 42 months and is often regarded as the gold standard for measuring the neurodevelopment of children. It covers five main domains of the child’s development including cognitive, language, motor, socio-emotional and adaptive behavior [33]. The two assessment tools of child development were administered by research staff from the NIN who were trained on the standardized assessment methods by the child development experts. They were selected by the trainers, based on the performance evaluations after a two-day training for the ASQ-3 and a four-week training for the Bayley scales. All the study staff who performed the outcome assessments were blinded to the study groups to which the subject was assigned.

### Anthropometry

Maternal and child anthropometric measurements were performed at the follow-up visit by the research staff of the NIN who were trained on standardized methods for collecting the measurements. The measurements included weight and height of the mother and child and the child’s mid-arm circumference at the follow-up visit. Weight was measured with subjects wearing light clothing and no shoes (Horse Head Brand TZ-120) and recorded to the nearest 0.1 kg. Standing height was measured using a stadiometer (Horse Head Brand TZ-120) and recorded to the nearest 0.1 cm. Child mid-upper arm circumference was measured from the upper left arm using a flexible, non-stretchable tape (Seca 201) and recorded to the nearest millimeter. BMI (kg/m^2^) was calculated from the measured weight and height. Weight-for-age and height-for-age were expressed as sex-age-specific z-scores using the WHO Child Growth Standards [34]. All the study staff who performed the anthropometric measurements were blinded to the study groups to which the subject was assigned.

### Sociodemographics and home environment

Sociodemographic information such as father’s education level, household income, smoking habits and alcohol consumption of the parents was obtained at the 30 months’ postpartum follow-up visit. A home environment questionnaire, adapted from the Home Observation for Measurement of the Environment Short Form (HOME-SF), was used to evaluate the cognitive stimulation of the child’s environment and the emotional relationship between mother and children [35].

### Sample size

The sample size was calculated based on the duration of ABF from birth prior to the study. A previous 30-month cohort study (n = 681) showed that the median duration of ABF was 9 months with an interquartile range (IQR) of 5 to 12.5 months [36]. Based on two-sample Wilcoxon rank-sum test with a two-tail test α of 5% with 80% power, it was estimated that at least 96 mother-child pairs would be required to detect a 20% difference in the mean duration of ABF between the intervention and control groups (POWER procedure, SAS Version 9.3, SAS Institute, Cary, NC, USA).

### Statistical analyses

Continuous variables were presented as mean ± standard deviation (SD) and categorical variables were presented as absolute numbers and percentages. Group difference was examined using Student's t-test or Wilcoxon rank-sum test for continuous variables, as appropriate. Categorical data between the groups were compared using Chi-squared test. Analysis of covariance (ANCOVA) was used as a primary analysis to assess the impact of intervention on breastfeeding duration and development outcomes, controlling for child’s age, gender, study site and other potential confounding variables such as household income, parents’ highest education level, smoking, mode of delivery, children’s anthropometrics, mother’s BMI, home environment score, etc. Confounding factors with a p-value below 0.3 in the univariate analysis were included in the multivariate analysis. The normality assumption was checked using quantile-quantile (q-q) plot and Shapiro-Wilk test that declared non-normal if found significant (p < 0.001). Wilcoxon rank-sum test was conducted if the residuals were non-normally distributed. Cox’s proportional Hazard Ratios (HR) and 95% confidence interval (CI) were estimated for discontinuing EBF. As a secondary analysis, multiple logistic regression models were used to estimate the Odds Ratios (OR) of getting above-median Bayley scores, controlling for study site, child age, gender and other covariates including sociodemographic, anthropometric and home environment factors. The OR estimates do not approximate risk ratios when outcomes are common.

The positive effects of the intervention on child development may be mediated through having better birth outcomes and prolonged EBF duration, which may, in turn, promote later cognitive and psychomotor development. Therefore, we conducted a regression-based mediation analysis [37] in which a possible mediator that was significantly associated with the intervention and a developmental outcome was added to the regression model. Changes in the coefficients for the intervention were then compared between models with and without the mediator. If the intervention is no longer significant when the mediator is included, the finding supports that the effect of intervention is fully mediated. If the association between intervention and development is attenuated but remains significant when the mediator is included, the finding supports partial mediation. Since birth weight and duration of EBF were significantly associated with the intervention and both may lie on the pathway between the intervention and child development, they were tested as potential mediators in the regression-based mediation analysis.

All the statistical analyses were performed using SAS (Version 9.3, SAS Institute, Cary, NC, USA). All statistical tests were two-sided. A p-value <0.05 was considered statistically significant and a p-value <0.10 was considered marginally significant.

## Results

### Parental and child characteristics

Four mother-child pairs were lost to follow-up in each study group (Fig 1). There were no children who were excluded from this study due to a diagnosis of development disorders. Table 1 summarizes socio-demographics and characteristics of the parents and children at the 30 months’ postpartum follow-up visit. All participant characteristics were comparable between the study groups (all p ≥ 0.1644). The mean age (SD) of the mothers at 30 months postpartum was 26.5 (2.6) and 26.8 (3.0) years for the intervention and control group, respectively. A majority of the parents had at least high school education (75.5% and 67.4% for mothers and fathers respectively) with an average annual household income of 128.15 × 10^6^ Vietnamese Dong (VND), which is about 5,645 USD. None of the mothers reported smoking during pregnancy and the follow-up until 30 months postpartum. All women reported to be either non-alcohol drinkers or having alcoholic drinks <1 day/month, except for one in the control group who reported daily consumption of alcohol. Only first-time mothers were recruited in the RCT; hence all children were the first live births. There were no significant differences in mothers’ BMI, children’s weight, height and mid-arm circumference at 30 months postpartum (all p ≥ 0.4455).

**Table 1:** Family sociodemographic and participant characteristics at the 30 months postpartum follow-up visit.

Characteristics	Intervention	Control	*P* value
**Mother-child pairs, n**	100	96	
**Child age (months), mean (SD)**	30.4 (0.7)	30.3 (0.6)	0.4338^a^
**Child gender, n (%)**			0.9766^c^
**Male**	55 (55.0)	53 (55.2)	
**Female**	45 (45.0)	43 (44.8)	
**Child weight (kg), mean (SD)**	12.34 (1.36)	12.30 (1.47)	0.8339^b^
**Child height (cm), mean (SD)**	88.24 (3.03)	88.24 (3.13)	0.9952^b^
**Child mid-arm circumference (cm), mean (SD)**	15.46 (1.23)	15.56 (1.23)	0.5638^b^
**Child weight for age z-score, mean (SD)**	-0.6 (0.9)	-0.6 (0.9)	0.7791^b^
**Child height for age z-score, mean (SD)**	-1.1 (0.8)	-1.1 (0.9)	0.9750^b^
**Mother age (years), mean (SD)**	26.5 (2.6)	26.8 (3.0)	0.9759^a^
**Mother BMI (kg/m^2^), mean (SD)**	20.5 (2.1)	20.8 (2.3)	0.4455^b^
**Annual household income (×10^6^ VND), n (%)**			0.4803^c^
**Poor (≤72)**	24 (24.0)	25 (26.0)	
**Near-poor (>72- ≤96)**	18 (18.0)	25 (26.0)	
**Average (>96- ≤120)**	31 (31.0)	25 (26.0)	
**Good or rich (>120)**	27 (27.0)	21 (21.9)	
**Mother education level, n (%)**			0.8708^c^
**Primary or secondary**	23 (23.0)	25 (26.0)	
**High school**	38 (38.0)	34 (35.4)	
**College/university or higher**	39 (39.0)	37 (38.5)	
**Father education level, n (%)**			0.1644^c^
**Primary or secondary**	26 (26.0)	36 (38.3)	
**High school**	44 (44.0)	32 (34.0)	
**College/university or higher**	30 (30.0)	26 (27.7)	
**Father alcohol consumption, n (%)**			0.2731^c^
**None**	34 (34.3)	24 (25.3)	
**<1 day/month**	5 (5.1)	11 (11.6)	
**1–3 days/month**	31 (31.3)	37 (39.0)	
**1–2 days/week**	22 (22.2)	17 (17.9)	
**≥3 days/week**	7 (7.1)	6 (6.3)	
**Father smoking, n (%)**			0.4154^c^
**Yes**	40 (40.4)	33 (34.7)	
**No**	59 (59.6)	62 (65.3)	
**Total smoking in household (sticks/day), mean (SD)**	4.3 (6.9)	3.8 (6.2)	0.5753^a^
**Food fortified using vegetable oil, n (%)**			0.9321^c^
**Yes**	92 (92.0)	88 (91.7)	
**No**	8 (8.0)	8 (8.33)	
**Home environment questionnaire score, mean (SD)**	6.2 (1.5)	6.5 (1.4)	0.1883^a^
**Child eating behavior questionnaire score, mean (SD)**	3.1 (0.4)	3.1 (0.5)	0.4809^a^

SD, standard deviation; VND, Vietnamese Dong, 1 US dollar is equivalent to 22681 VMD.

^a^ P-value from Wilcoxon rank-sum test.

^b^ P-value from two-sample t-test.

^c^ P-value from Chi-squared test.

### Breastfeeding practices

**Fig 2:**
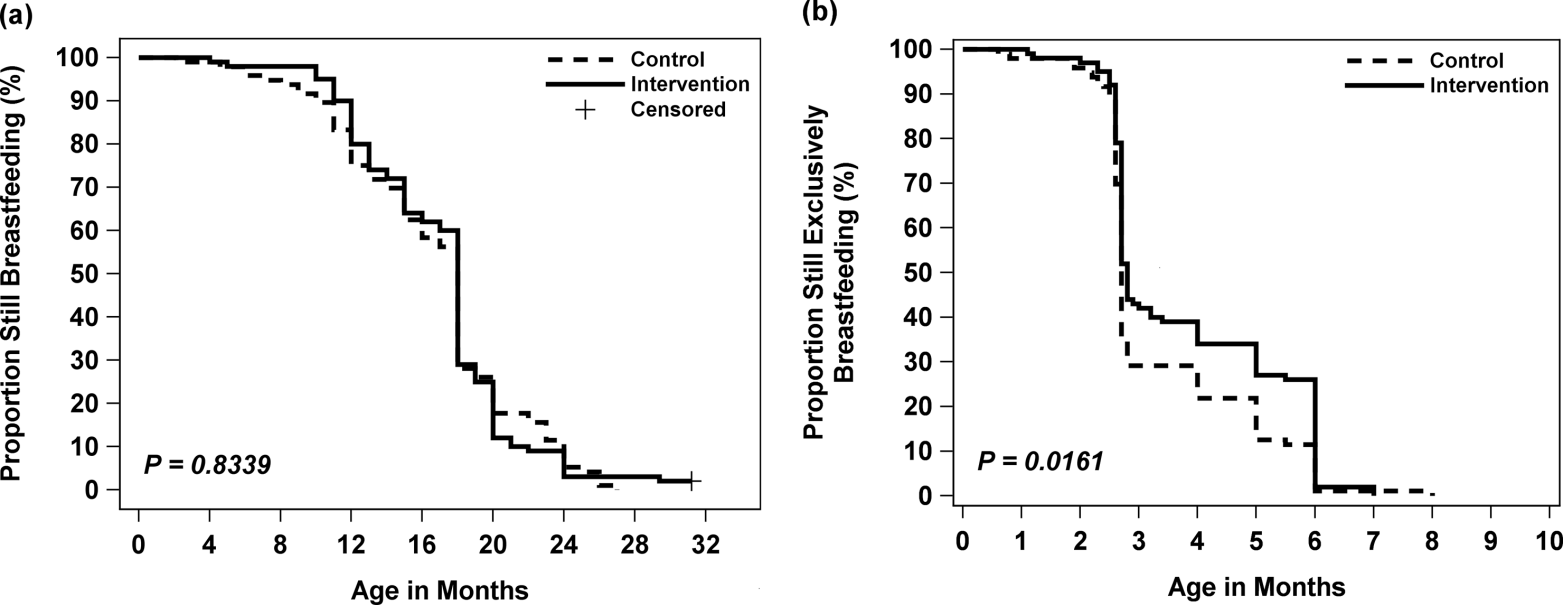
Kaplan-Meier survival curves for duration of (a) any breastfeeding duration and (b) exclusive breastfeeding by treatment. - - - - - Control, ^_________^ Intervention, + Censored.

Fig 2. illustrates the Kaplan-Meier curves for duration of ABF (Fig 2A) and EBF (Fig 2B). No significant difference was observed for time to stop ABF (p = 0.8339), whereas time to stop EBF between the intervention and control group differed significantly (p = 0.0161) whereas no significant difference was observed for time to stop ABF (p = 0.8339). The percentage of EBF dropped dramatically around 3 months of age and continued to decrease steadily thereafter in both groups (Fig 2B). As shown in Table 2, there were no significant differences in ABF at 1 year (p = 0.1690) and at 2 years (p = 0.5698) between the groups. More than 80% infants were given ABF at 12 months of age, while ABF at 24 months of age dropped to less than 12% in both groups (Table 2). Because the data of ABF at 30 months postpartum were censored for two children, a sensitivity analysis was performed by excluding these two children, which did not affect the results. The intervention group had a significantly higher percentage of EBF than the control at 3 months postpartum (43.0% vs. 29.2% respectively, p = 0.0440). This remained significantly higher in the intervention group compared with the control at 6 months postpartum (26.0% vs. 11.5% respectively, p = 0.0093). A multiple Cox regression of breastfeeding duration confirmed the protective effect of the intervention in reducing the risk of discontinuing EBF (Table 3). Compared with the control group, the intervention group had an adjusted HR of 0.67 (95% CI: 0.50–0.90) for discontinuing EBF after controlling for potential confounders. This indicates that mothers in the intervention group were 33% less likely to stop EBF than those in the control group.

**Table 2:** Maternal breastfeeding and child feeding practices.

Variable	Intervention (*n* = 100)	Control (*n* = 96)	*P* value
Duration of exclusive breastfeeding (weeks), mean (SD)	16.5 (6.8)	14.5 (5.9)	0.0172^a^
Time to introduce complementary foods (weeks), mean (SD)	24.5 (4.6)	24.5 (5.6)	0.9392^a^
Duration of any breastfeeding (weeks), mean (SD)	73.9 (20.1)	72.4 (22.4)	0.4334^a^
Exclusive breastfeeding at 3 months, n (%)	43 (43.0)	28 (29.2)	0.0440^b^
Exclusive breastfeeding at 6 months, n (%)	26 (26.0)	11 (11.5)	0.0093^b^
Introduction of solids and semi-solids at 6 months, n (%)	44 (44.0)	48 (50.0)	0.4001^b^
Any breastfeeding at 1 year, n (%)	90 (90.0)	80 (83.3)	0.1690^b^
Any breastfeeding at 2 year, n (%)	9 (9.0)	11 (11.5)	0.5698^b^

^a^ P-value from analysis of covariance (ANCOVA) controlling for study site, child age, gender and other covariates including education, smoking, mode of delivery, initiation of breastfeeding, milk adequacy, mother’s BMI and subsequent pregnancies.

^b^ P-value from Chi-squared test.

**Table 3:** Results of Cox’s proportional hazards models (*HR*s, 95% CI) for discontinuing exclusive breastfeeding.

Variable	Adjusted *HR*	95% CI	*P* value
**Treatment**			
**Intervention**	0.67	(0.50, 0.90)	0.0071
**Control**	1	Reference	
**Child age (months)**	1.16	(0.83, 1.63)	0.3774
**Child gender**			
**Female**	1.59	(1.19, 2.14)	0.0020
**Male**	1	Reference	
**Study sites**			
**Ninh Binh**	0.50	(0.32, 0.80)	0.0035
**Ha Nam**	0.96	(0.61, 1.52)	0.8690
**Hai Phong**	0.94	(0.64, 1.39)	0.7710
**Thai Nguyen**	1	Reference	
**Mode of delivery**			
**Cesarean section**	2.24	(1.58, 3.19)	< .0001
**Natural**	1	Reference	
**Initiated breastfeeding in 1 hour**			
**Yes**	0.66	(0.43, 1.01)	0.0547
**No**	1	Reference	

*HR*, Hazard Ratio; *n* = 196.

The average age when complementary foods were introduced was around 24.5 weeks for both groups (p = 0.9392) (Table 2). Premature introduction of solids and semi-solids during the first six months of life was observed among some infants. By the age of 6 months, 50% (48/96) of the infants in the control group had been introduced to (semi-)solid foods, compared to 44% (44/100) in the intervention (p = 0.4001).

### Effects of intervention on child development

Mean developmental scores of children at 30 months in the intervention and control groups are shown in Table 4. Children born to mothers who received the intervention scored better on the Bayley-III composite scores in the domains of cognitive (p = 0.0498) and motor (p = 0.0422) functions, as well as a tendency toward higher composite score for social-emotional function (p = 0.0513), compared to those born to mothers received standard care. No significant between-group differences were observed for the Bayley language (p = 0.7329) and general adaptive behavior (p = 0.5399) scores.

**Table 4:** Development scores from ASQ-3 and Bayley-III at 30 months of age by study groups.

Development Score	Mean (SD)		Median (IQR)		*P* value
	Intervention (*n* = 100)	Control (*n* = 96)	Intervention (*n* = 100)	Control (*n* = 96)	
**ASQ-3 score**					
**Problem solving**	40.7 (8.6)	41.1 (7.6)	40.0 (35, 45)	40.0 (37.5, 45.0)	0.7688^a^
**Communication**	55.9 (6.9)	56.2 (5.4)	60.0 (55.0, 60.0)	60.0 (55.0, 60.0)	0.8932^b^
**Gross motor**	51.4 (6.8)	50.4 (7.1)	50.0 (50.0, 55.0)	50.0 (45.0, 55.0)	0.2788^b^
**Fine motor**	49.5 (11.5)	46.6 (12.4)	55.0 (40.5, 60.0)	50.0 (40.0, 57.5)	0.0705^b^
**Personal-social**	49.2 (6.7)	48.4 (7.4)	50.0 (45.0, 55.0)	50.0 (45.0, 50.0)	0.6144^b^
**Total score**	246.6 (27.7)	242.7 (25.9)	255.0 (232.5, 265.0)	245.0 (230.5, 260.0)	0.1140^b^
**Bayley-III composite score**					
**Cognitive**	100.3 (9.1)	98.3 (9.7)	100.0 (95.0, 105.0)	95.0 (90.0, 100.0)	0.0498^b^
**Language**	103.1 (9.7)	102.6 (9.8)	103.0 (97.0, 107.5)	103.0 (97.0, 109.0)	0.7329^a^
**Motor**	106.9 (13.8)	103.1 (11.7)	110.0 (97.0, 115.0)	103.0 (94.0, 111.0)	0.0422^a^
**Social-emotional**	86.7 (12.3)	83.5 (13.4)	85.0 (80.0, 95.0)	80.0 (75.0, 87.5)	0.0513^b^
**General adaptive**	90.8 (14.8)	90.1 (14.8)	93.0 (80.0, 100.5)	93.0 (81.5, 98.5)	0.5399^a^
**Bayley-III scaled score**					
**Receptive communication**	10.8 (2.0)	10.8 (2.1)	11.0 (10.0, 11.0)	11.0 (10.0, 12.0)	0.8975^b^
**Expressive communication**	10.2 (1.7)	10.1 (1.5)	10.0 (9.0, 11.0)	10.0 (9.0, 11.0)	0.5701^b^
**Gross motor**	10.6 (2.9)	9.8 (2.1)	10.0 (9.0, 12.0)	10.0 (8.0, 11.0)	0.0534^a^
**Fine motor**	11.7 (2.9)	11.1 (2.8)	11.0 (9.0, 14.0)	11.0 (9.0, 13.0)	0.1848^a^

^a^ P-value from analysis of covariance (ANCOVA) controlling for study site, child age, gender and other covariates including sociodemographic, anthropometric and home environment factors

^b^ P-value from Wilcoxon rank-sum test for scores with non-normal residuals.

Similar effects were shown in the multiple logistic regression using dichotomized outcomes at the median values of developmental scores (Table 5). Compared to the control, children in the intervention group had significantly higher odds of achieving a Bayley-III score above the median in the domains of cognition (OR: 1.81, 95% CI 1.01–3.26, p = 0.0478) and motor (OR: 2.83, 95% CI 1.48–5.38, p = 0.0016).

**Table 5:** Odds Ratios (and 95% confidence intervals) of getting an above the median score of neurodevelopment at 30 months of age among children in the intervention group compared to the control.

Development	Intervention *(N*_*a*_*/N*_*b*_*)*	Control *(N*_*a*_*/N*_*b*_*)*	Adjusted *OR* (95% CI)	𝑃 value
**ASQ-3 score**				
**Problem solving**	42/58	40/56	0.99 (0.56, 1.78)	0.9815
**Communication**	55/45	53/43	1.00 (0.57, 1.76)	0.9951
**Gross motor**	40/60	34/62	1.20 (0.66, 2.20)	0.5557
**Fine motor**	52/48	37/59	1.82 (0.98, 3.36)	0.0565
**Personal-social**	28/72	22/74	1.32 (0.68, 2.57)	0.4174
**Total score**	53/47	39/57	1.63 (0.90, 2.93)	0.1051
**Bayley-III composite score**				
**Cognitive**	59/41	44/52	1.81 (1.01, 3.26)	0.0478
**Language**	42/58	40/56	1.04 (0.58, 1.88)	0.8986
**Motor**	52/48	31/65	2.83 (1.48, 5.38)	0.0016
**Social-emotional**	57/43	47/49	1.53 (0.84, 2.76)	0.1617
**General adaptive**	49/51	45/51	1.24 (0.68, 2.24)	0.4805

P-value from logistic regression controlling for study site, child age, gender and other covariates including sociodemographic, anthropometric and home environment factors. Development scores were dichotomized using the median. *Na*: number of children with a developmental score above the median; *Nb*: number of children with a developmental score below the median. *OR*, Odds Ratio.

When the developmental performance was assessed using ASQ-3, no significant differences were detected in all 5 domains of child development, except for a tendency toward higher fine motor score in the intervention group compared to the control group (mean score: 49.5 vs. 46.6, p = 0.0705) (Table 4). In the multiple logistic regression where ASQ-3 scores were dichotomized, children in the intervention group tend to have higher odds of scoring above the median in fine motor function (OR: 1.82, 95% CI 0.98–3.36, p = 0.0565) (Table 5).

## Discussion

The present study compared the long-term effects of a maternal milk supplementation (MMS) in conjunction with a breastfeeding support program on breastfeeding practices and child neurodevelopment outcomes at 30 months old. Our results showed minimal differences in ABF between the intervention and control groups. However, the intervention had sustained effects on post-intervention breastfeeding practices including prolonged EBF duration, increased EBF rate at 6 months and reduced risk of early EBF cessation in Vietnamese mothers. In addition, the intervention was associated with improved cognitive, motor, and socio-emotional development in the offspring at 30 months old.

Mothers who received MMS and breastfeeding support during late pregnancy and early lactation were found to have a prolonged duration of ABF (74 weeks versus 72 weeks), although not statistically significant. Furthermore, the intervention significantly improved long-term EBF behavior in our study. This finding is especially important for Vietnam, a country where breastfeeding practices remain suboptimal [38,39,40]. Despite various breastfeeding promotional activities over the years such as restrictions on formula advertising and increased maternity leave from four months to six months, the national prevalence of EBF at six months of age and ABF after 1 years remains low [38,39,41]. In our study, ABF rate at 2 years of age dropped to less than 12% and the percentage of infants who were still exclusively breastfed at 6 months of age was only 11.5% for the control group. Both rates are lower than the national average of 22.1% and 19.6% for ABF and EBF, respectively in 2010 reported by the NIN [41]. The lower breastfeeding rates in our study are likely due to the differences in sociodemographic and environmental factors in our study population compared to those included in the national survey. For example, the subjects in our study comprised only first-time mothers from four northern provinces in Vietnam, which may not represent the entire general population in the country. While ABF rate was not significantly improved by our intervention, EBF rate at 6 months in the intervention group was increased to 26.0%, which is slightly higher than the nationally reported EBF rate. This finding suggests that the intervention regimen consisting of MNS and breastfeeding support until 12 weeks postpartum may help establish a favorable breastfeeding behavior and reinforce good breastfeeding practices among the first-time mothers during perinatal and early postpartum periods. This may in turn help sustain the EBF practice until 6 months postpartum after the intervention was completed. To our knowledge, the present study is the first to examine post-intervention effects of an intervention providing both MMS and breastfeeding support on long-term breastfeeding practices. Given that EBF rate in Vietnam remains far below the national target of 35% by 2020 [42], promoting breastfeeding through MMS and breastfeeding support could be an effective strategy to enhance EBF practices.

Further to this, there is limited evidence that has examined the effect of breastfeeding support and dietary intervention on both EBF and ABF. Most previous studies in this area focused on either EBF or ABF as one of the outcomes [13,43,44]. In addition, previous research was designed to investigate the effect of breastfeeding support [45] or dietary intervention [46] alone on EBF and ABF. The present study extends our knowledge from the aforementioned studies as it examined the effect of a maternal milk supplementation from the last trimester to 12 weeks postpartum in conjunction with a breastfeeding support program on both EBF and ABF. Our results suggest that the intervention may have an immediate effect on continuing EBF than ABF. Further studies are warranted to confirm this finding.

The present study showed that the intervention during perinatal period had minimal influence on child growth in terms of weight, height, and mid-arm circumference at 30 months old. In a double-blind controlled trial among 747 nutritionally at-risk Indonesian women, children born to mothers who received a high-energy supplement (465 kcal, 7.1 g protein) daily in the last trimester of pregnancy showed better growth in weight and height than those born to mothers who were given a low energy supplement (52 kcal, 6.2 g protein) [28,47]. However, the between-group difference in weight was only significant until 24 months, whereas height was significantly higher in the intervention group throughout the first 5 years [47]. This is inconsistent with our observation of null difference, in which the children’s anthropometrics were measured at around 30 months. The discrepancy may be due to the much higher energy supplementation in the Indonesia study (465 kcal) compared to the present study (252 kcal). It may also be due to the different characteristics of the target population. For example, mothers in the Indonesia trial were short and lean who had at least one child before the intervention, whereas the mothers in our study were all first-time mothers and most of them had normal pre-pregnant BMI. In addition, only the children of mothers who had high compliance were followed-up in the Indonesia study whereas our results were based on intention-to-treat analysis.

The intervention in our study was significantly associated with improved cognition, motor, and social-emotional behavior assessed using Bayley-III in the offspring at age 30 months. Two potential mechanisms, not mutually exclusive, have been suggested to account for the neurodevelopmental impact following nutrition supplementation early in life: (1) directly affecting brain development and function by increasing nutrient supply and (2) indirectly affecting children’s experiences and behavior by interacting with the environment [19].

First, both macro- and micro-nutrients provided in our intervention improved maternal nutritional adequacy, which may have supported the structural and functional development of the brain during gestation and infancy. Evidence from animal and human research suggested that nutrient deficiency during pregnancy and lactation adversely affected neuronal cell growth, pruning, myelination, and nervous system formation within regions of the brain, all of which resulted in impaired motor functioning, memory, learning, and higher order cognition [19,48]. Many nutrients, including those relevant to infant neurological development, such as vitamin A, B, folate, iodine, selenium and fatty acids, have been found to be increased in the breast milk of mothers with better maternal nutrition [49,50], which will provide a higher nutrient supply to breastfed infants to support growth and development during the postnatal period. This potential mechanism is supported by results from the mediation analyses. In multiple regressions, further adjustment for birth weight attenuated the association between intervention and Bayley-III cognitive, motor and social-emotional functions (data not shown), suggesting that the benefits of the intervention on child development may be partially attributed to the positive effect of prenatal nutrition supplementation on birth outcomes.

Second, breastfeeding has long been suggested to positively influence the maternal bond and/or infant attachment, although this hypothesis has not been well-supported largely because of limited data in this area [51]. In our study, the intervention significantly increased the duration and rate of EBF and reduced the risk of EBF cessation. However, the duration of EBF was not associated with developmental scores. Therefore, the positive effect of the intervention on developmental outcomes was unlikely to be mediated by prolonged duration of EBF. These results suggest that the prenatal nutritional supplementation may have played a more important role than the postnatal breastfeeding support in promoting the neurodevelopment in this child population.

Our hypothesis is partially supported by the few available intervention trials conducted in the 1960s that involved nutritional supplementation during pregnancy and lactation. In a double-blind RCT in 225 Taiwanese women, infants born to mothers who consumed a high calorie-protein liquid supplement (800 kcal, 40g protein/day) had significantly higher Bayley-II motor score at 8 months than those born to mothers who consumed a low-calorie no-protein placebo (≤80 kcal/day) [24]. However, no significant group differences were found on the Bayley-II mental development assessed at 8 months of age and intelligence quotient (IQ) at the age of 5 years [24,29]. Another RCT in 768 malnourished women in the New York City found significant improvements of daily high protein-energy supplementation (470 kcal, 40 g protein) during pregnancy on three psychological measures at 1 year of age, including visual habituation, visual dishabituation, and mean length of free play episodes, compared to the group received no supplementation [12]. However, the offspring showed no improvements in the scores of Bayley motor and mental development, object permanence or sophistication of play at 12 months of age [25]. The inconsistent results may in part be explained by differences in study design, population group, type, dose and duration of supplementation, age of the child and timing for assessing child development outcomes (i.e. post-intervention or during the intervention).

Brain structures and functioning develop sequentially. Structurally, the cerebellum (important for motor control) develops from early prenatal life and continues to the postnatal period while the striatum (coordinates multiple aspects of cognition) develops rapidly during the late gestational and early postnatal period [48,52]. Myelination and neural connectivity across diffusely distributed systems largely occur postnatally, which has a great impact on intelligence quotient [53]. Functionally, sensory pathways like those for the basic vision and hearing are the first to develop in the first year of a child’s life, followed by early language skills and then by cognitive functions that form over the years into adulthood [54]. Thus, there may be differences by the timing of nutrition supplementation and the age of development assessment as well as domains of functioning evaluated. While the Taiwan trial showed that MMS improved children’s motor but not mental development at 8 months of age, our study showed positive effects on both motor and cognitive development at 30 months of age. This might be because cognitive assessment before age 2 years is not sensitive enough to detect the difference. In our study, MMS was given starting from the last trimester until 12 weeks postpartum, which may only affect functions in the regions of the brain that develop during this period such as the striatum that coordinates cognition. However, our intervention had no effect on children’s general adaptive behavior measured at 30 months of age. This assessment age may be too early for any improvement to occur as children tend to gain adaptive skills through age and experience and most children develop basic self-help skills by age 5 or 6. These results together with previous studies support the hypothesis that nutritional supplementation to mothers during pregnancy and breastfeeding, with or without breastfeeding support, improves the neurodevelopment ability of the offspring in early childhood.

This study has several strengths, including its multicenter structure, randomization of treatment, high participation rate, blinding of the outcome assessors, objective assessment of child development, and the thorough consideration of potential confounders.

Nevertheless, our study has several limitations. First, this was an observational follow-up study and part of the data was collected retrospectively. Information on breastfeeding practice and child feeding behavior at 30 months postpartum was therefore subjected to recall bias. However, the literature suggests that maternal recall provides reasonably valid and reliable estimates of breastfeeding initiation and duration, especially when the length of the recall period is short (≤3 years), which is the timeframe of collecting breastfeeding information in our study [55]. On the other hand, validity and reliability of maternal recall for the age at introduction of food and fluids other than breast milk are less satisfactory [55,56].

Second, while we have used two different development assessment tools including ASQ-3 and Bayley-III, the two scales showed differences in detecting some developmental domains. The discrepancy is consistent with a previous study which had compared the validity of the two scales and found poor agreement [57]. ASQ-3 is a child developmental screening tool based on questionnaires designed to be completed by parents and caregivers. On the other hand, in Bayley-III, cognition, language, and motor performance are assessed through direct observation of the child while social-emotional and adaptive behavior is assessed through questionnaires to be completed by the main caregiver. Although parents’ view of the developmental status of their infants has been considered appropriate, it is subjective and prone to bias. This may partially explain why the cognitive development score using Bayley-III was significant in our study but the problem-solving score using ASQ-3 was not significant.

Third, although both Bayley-III and ASQ-3 were translated into Vietnamese and used in previous studies in Vietnam [58,59], neither has been adapted and validated for use in the Vietnam setting. Furthermore, there is a potential lack of statistical power for development outcomes in our study since the original sample size calculation was based on the primary outcome variable, i.e. the duration of ABF.

## Conclusions

In summary, maternal milk supplementation from the last trimester to the first 12 weeks postpartum, coupled with a breastfeeding support program was significantly associated with prolonged EBF duration, increased EBF rate at 6 months, and reduced risk of early EBF cessation. The intervention also significantly improved child neurodevelopment in the domains of cognitive and motor functions at 30 months of age, when compared with the current standard care. The benefits of the maternal intervention on child neurodevelopment were partially mediated by higher birth weight but not EBF duration. This suggests that the prenatal nutritional supplementation may have played a more important role than the additional breastfeeding support given during the postnatal period in promoting neurodevelopment of the offspring. Future studies with a larger sample size are needed to examine the longer-term impacts of maternal nutritional supplementation on child growth and development.

## Supporting information

S1 Table List of regression models. (DOCX)
