# Supplementary material for: Impact of maternal nutritional supplementation in conjunction with a breastfeeding support program during the last trimester to 12 weeks postpartum on breastfeeding practices and child development at 30 months old
Source: PLoS One. 2018 Jul 16;13(7):e0200519. doi: 10.1371/journal.pone.0200519 (PMC6047798; doi:10.1371/journal.pone.0200519)
Supplement: S1 Table — (DOCX) [file pone.0200519.s001.docx]

Table S1. List of regression models

| **Methods** | **Regression Models** |
| --- | --- |
| ANCOVA for Table 2 | ABF duration = child age + gender + site + treatment + gestational age + mother’s pre-pregnancy BMI + mother had any subsequent pregnancies  EBF duration = child age + gender + site + treatment + initiation of breastfeeding (in minutes) + mode of delivery + mother weight + home environment score  Time to introduce complementary foods = child age + gender + site + treatment + education + smoking + mode of delivery |
| ANCOVA for Table 4 | ASQ Problem Solving score = child age + gender + site + treatment + education + smoking  Bayley Language composite score = child age + gender + site + treatment + length for age z-score  Bayley Motor composite score = child age + gender + site + treatment + education + home environment score  Bayley General Adaptive composite score = child age + gender + site + treatment + education + home environment score + mother subsequent pregnancies + alcohol + child length for age z-score  Bayley Gross Motor scaled score = child age + gender + site + treatment + education + home environment score + mother had any subsequent pregnancies + child length for age z score  Bayley Fine Motor scaled score = child age + gender + site + treatment + income + child mid arm circumference |
| Logistic Regression for Table 5 | Logit (ASQ Problem Solving) = child age + gender + site + treatment + use of vegetable oil during food preparation + education  Logit (ASQ Communication) = child age + gender + site + treatment + use of vegetable oil during food preparation  Logit (ASQ Gross Motor) = child age + gender + site + treatment + income + alcohol  Logit (ASQ Fine Motor) = child age + gender + site + treatment + education + use of vegetable oil during food preparation + mother BMI  Logit (ASQ Personal-Social) = child age + gender + site + treatment + use of vegetable oil during food preparation  Logit (ASQ Total Score) = child age + gender + site + treatment + education  Logit (Bayley Cognitive composite score) = child age + gender + site + treatment + income + education + use of vegetable oil + mother BMI  Logit (Bayley Language composite score) = child age + gender + site + treatment + income  Logit (Bayley Motor composite score) = child age + gender + site + treatment + education + home environment score  Logit (Bayley Social-Emotional composite score) = child age + gender + site + treatment + home environment score + mother had any subsequent pregnancies + alcohol consumption + income  Logit (Bayley General Adaptive composite score) = child age + gender + site + home environment score + mother had any subsequent pregnancies + income |
